# Novel insights and therapeutic approaches in secondary AML

**DOI:** 10.3389/fonc.2024.1400461

**Published:** 2024-07-29

**Authors:** Giovanni Marconi, Michela Rondoni, Beatrice Anna Zannetti, Irene Zacheo, Davide Nappi, Agnese Mattei, Serena Rocchi, Francesco Lanza

**Affiliations:** ^1^ Hematology Unit and Romagna Transplant Network, Hospital of Ravenna, University of Bologna, Ravenna, Italy; ^2^ Hematology Unit and Romagna Transplant Network, Hospital of Ravenna, Ravenna, Italy; ^3^ IRCCS Istituto Romagnolo per lo Studio dei Tumori (IRST) “Dino Amadori”, Meldola, Italy

**Keywords:** acute myeloid leukemia, AML, secondary AML, myelodysplasia-related, chemotherapy-related, AML genetics, novel therapies

## Abstract

Secondary acute myeloid leukemia (sAML) presents as a complex and multifaceted ensemble of disorders, positioning itself as both a challenge and an intriguing frontier within hematologic oncology. Its origins are diverse, stemming from antecedent hematologic conditions, germline predisposing mutations, or the sequelae of cytotoxic therapies, and its development is driven by intricate genetic and epigenetic modifications. This complexity necessitates a diverse array of therapeutic strategies, each meticulously tailored to address the distinctive challenges sAML introduces. Such strategies require a personalized approach, considering the variegated clinical backgrounds of patients and the inherent intricacies of the disease. Allogeneic stem cell transplantation stands as a cornerstone, offering the potential for curative outcomes. This is complemented by the emergence of innovative treatments such as CPX-351, venetoclax, and glasdegib, which have demonstrated promising results in enhancing prognosis. The evolving landscape of sAML treatment underscores the importance of continued research and innovation in the field, aiming not only to improve patient outcomes but also to deepen our understanding of the disease’s biological underpinnings, thereby illuminating pathways toward more effective and individualized therapies.

## 1 Introduction

Acute myeloid leukemia (AML) represents a heterogeneous group of hematologic malignancies characterized by the clonal expansion of myeloid precursors with impaired differentiation and proliferation. Among these, secondary AML (sAML) emerges as a distinct category, encompassing cases that evolve from a preexisting hematologic disorder or as a consequence of cytotoxic therapy (1).

Globally, the incidence of AML varies, but it is generally considered a disease of older adults, with the median age at diagnosis around 68 years. In the United States, AML accounts for approximately 1.1% of all cancers, with an estimated 20,240 new cases and approximately 11,400 deaths in 2022. The age-adjusted incidence rate is approximately 4.3 per 100,000 persons per year. AML incidence increases significantly with age, rising sharply in individuals over 60 years old. The disease is slightly more common in men than in women and has higher incidence rates among Caucasians compared with other ethnic groups (2, 3). sAML occurs in approximately 10%–20% of all AML cases and is often associated with poorer prognosis compared with *de novo* AML. It typically develops as a late complication of cytotoxic chemotherapy or radiation therapy used to treat other cancers, such as breast or prostate cancer, or following exposure to environmental carcinogens like benzene. The latency period between the primary treatment and the onset of sAML can range from a few months to several years, averaging approximately 3–5 years. This form of leukemia often exhibits multidrug resistance at diagnosis, making it more challenging to treat (4). The prognosis for sAML depends on various factors, including patient age, comorbidities, previous exposure to chemotherapy and radiation for other cancers, cytogenetics, access to transplant, and molecular abnormalities. Overall survival rates have improved modestly over the past few decades and remain poor (5, 6).

This review endeavors to dissect the intricate landscape of sAML, underscoring its etiology, genetic landscape, clinical implications, and the evolving therapeutic paradigms aimed at addressing its unique challenges. The pathogenesis of sAML is intricately linked to its precursor states, with genetic and epigenetic alterations playing pivotal roles in its evolution.

The diagnostic landscape of sAML has been refined by the integration of molecular and cytogenetic insights, with recent classifications from the World Health Organization (WHO22) and the International Consensus Classification (ICC22) offering frameworks that reflect the disease’s heterogeneity and its prognostic implications (7, 8). Treatment strategies for sAML are challenged by the disease’s inherent complexity and the patients’ diverse clinical backgrounds. Allogeneic stem cell transplantation (allo-SCT) remains a cornerstone for potentially curative therapy, yet the approach is tailored based on individual patient factors, reflecting the personalized medicine ethos increasingly adopted in sAML management (9).

In sum, sAML represents a paradigm of the challenges and opportunities within hematologic oncology, embodying the need for a deep understanding of disease biology, a nuanced approach to classification and diagnosis, and a personalized strategy for therapeutic intervention. This review aims to elucidate the current understanding of sAML, from its etiological factors to the latest in therapeutic developments, highlighting the ongoing journey toward improved patient outcomes in this complex disease landscape.

## 2 Definition of secondary AML

AML encompasses a range of aggressive myeloid neoplasms that develop stochastically or due to certain known predisposing factors (1). sAML comprehends any non-stochastic case of AML and specifically refers to AML that arises because of certain predisposing factors; these factors include previous treatment with chemotherapy or radiation (therapy-related AML), a history of a myeloid neoplasm such as myelodysplastic neoplasms (MDS), i.e., AML secondary to MDS, or a genetic predisposition that increases the risk of developing AML, i.e., AML secondary to germline predisposition (7, 8).

### 2.1 AML secondary to MDS or other myeloid neoplasms

AML secondary to MDS or other myeloid neoplasms refers to AML that evolves from previously diagnosed neoplasms (7, 8). This transition is marked by an increase in myeloblasts in the bone marrow or blood, reflecting a progression from a primarily dysplastic disorder to an acute leukemia. The classification and diagnostic criteria for these cases have been refined to better reflect their unique biological and prognostic characteristics. The process of transformation into AML is known to be a stepwise acquisition of alterations with a disease natural history that begin with myelodysplasia and end with the aggressive transformation (10, 11). Of note, the definition of myelodysplasia-related (MR) alteration is becoming very important in clinical practice, and MR-AML includes diseases with genetic and cytogenetic alterations related to dysplasia. This characterization defines a subgroup of biologically defined newly diagnosed AML that has prognosis and characteristics similar to patients who have AML raised after MDS (7, 8).

### 2.2 AML secondary to chemotherapy

AML secondary to chemotherapy, also known as therapy-related AML (tAML), is a subtype that develops as a direct consequence of mutational events induced by cytotoxic therapy, such as chemotherapy (e.g., platinum or alkylating agents) or radiation (12). However, evidence demonstrated that a predisposition deriving from preexistent clonal hematopoiesis of indeterminate potential (CHIP) may contribute to disease development together or independently of chemotherapy (13–16). Most of tAML cases are associated with adverse genetic lesions and show a high frequency of abnormal karyotypes. The latency period between the initial therapy and the onset of AML typically ranges from 5 to 7 years for cases related to alkylating agents or radiation and is characterized by a predisposition to MDS and frequent chromosomal abnormalities (4, 17). Furthermore, this particular set of patients is characterized by comorbidities and frailties related to previous therapy exposure, which often make their treatment particularly difficult; there are significant evidence suggesting that in this subset, toxicity and non-relapse mortality are a relevant clinical problem (4).

### 2.3 AML secondary to germline predisposition

AML secondary to germline predisposition encompasses cases where individuals have an inherited genetic predisposition to hematopoietic malignancies, including AML (7, 8). This predisposition is due to mutations in genes that are critical for DNA repair, cell cycle regulation, or hematopoiesis (18–20). Identifying such predispositions is crucial for patient management, especially in the context of allogeneic hematopoietic cell transplantation (HCT) and surveillance strategies for the patient and their relatives (20). Germline predispositions can drive hematopoietic malignancies at any age, with some alleles being more prevalent in older individuals (21). Germline predisposition to leukemia is defined differently in WHO22 and ICC22 s (**Supplementary Table S1**); particularly, ICC22 also includes genetic diseases that confer a risk of a wide variety of solid and blood cancers even if leukemia is not within the most common manifestations, and a list of emerging genetic predispositions (7, 8). The clinical implications of recognizing germline predispositions to hematopoietic malignancies have become increasingly important for personalized patient management and family health surveillance. Recognizing these predispositions is particularly crucial when considering allogeneic hematopoietic cell transplantation as a treatment option. The identification of germline risk alleles also informs health surveillance strategies for both the patients and their relatives who might carry the same genetic variants. In such cases, identifying germline risk alleles is crucial as it influences donor selection, especially to avoid using donors who may carry deleterious variants such as those found in RUNX1 and CEBPA genes, which are known to be associated with poor transplant outcomes. Moreover, early genetic testing plays a pivotal role in guiding the treatment of other family members who might be at risk, ensuring that preventive measures and monitoring are implemented timely (22, 23).

In clinical practice, the recognition of germline predispositions to hematopoietic malignancies necessitates specific indicators for testing. Key clinical features prompting the consideration of germline testing include a personal history of multiple cancers, with at least one being a hematopoietic malignancy; a personal or familial history of early-onset cancers (diagnosed at age 50 or younger); the detection of deleterious gene variants during tumor profiling that persist during remission, suggesting a germline origin; and the diagnosis of a hematopoietic malignancy at an unusually early age, such as myelodysplastic syndromes diagnosed in patients younger than 40 years. Furthermore, the presence of a variant in tissues unlikely to undergo somatic mutations, such as cultured skin fibroblasts or hair follicles, or its detection in multiple relatives, strengthens the case for a germline origin (9, 23). These criteria are vital for clinicians to identify patients who may benefit from germline testing, thereby facilitating targeted surveillance and management strategies for both the patients and their at-risk relatives.

Virtually, all patients diagnosed with hematopoietic malignancies should be considered for germline testing, regardless of age, due to the potential for some alleles, like those in DDX41, to drive malignancies in older age (9, 20). The new frontier in the field will be the assessment of the impact of somatic polymorphism on leukemia risk; we previously identified a significant risk locus for leukemia at 11q13.2 (rs4930561; KMT5B) (24), and there is increasing evidence of germline predisposition to clonal hematopoiesis (25, 26).

### 2.4 WHO22 and ICC22 classification of sAML

A proper diagnosis of sAML highlights the importance of considering the patient’s treatment history, genetic background, and the presence of preexisting hematologic conditions in diagnosis and management. Unfortunately, the two available classifications present significant differences in sAML-related definitions, thus posing difficulties in reaching homogeneity in this sector (27). The WHO22 and ICC22 both offer frameworks for classifying myeloid neoplasms, including sAML, with a strong emphasis on genetic abnormalities and their impact on disease phenotype and outcome. ICC22 is notable for its hierarchical structure, where genetic aberrations are prioritized in defining AML disease classification. Additional predisposing features such as therapy-related, prior myelodysplastic syndrome (MDS) or MDS/myeloproliferative neoplasm (MPN), and germline predisposition are maintained as “anamnestic qualifier” and appended as qualifiers to the primary diagnosis (8). In WHO22 classification, myeloid neoplasms that arise secondary to exposure to cytotoxic therapy or germline predisposition are grouped in a separate category. AML transformation of MPN is retained in the MPN category, whereas AML transformation of MDS and MDS/MPN is kept under AML-MR (7).

The main difference between the WHO22 and ICC22 classifications lies in the approach to defining and classifying AML, with ICC22 focusing more on genetic aberrations and using a hierarchical structure that emphasizes genetic characteristics over clinical history for classification (**Table 1**). This shift highlights the importance of genetic profiling in diagnosing and treating AML, including secondary AML, and reflects a move toward more personalized medicine approaches in hematology. Both the WHO22 and ICC22 classifications cover the broad spectrum of secondary AML, recognizing the impact of therapy-related factors, prior myeloid neoplasms, and germline predispositions. However, the ICC’s more detailed approach to classifying AML based on genetic aberrations and its inclusion of specific diagnostic qualifiers might result in a more nuanced identification of sAML subtypes (28). Both ICC22 and WHO22 acknowledge that bone marrow blast cell number at diagnosis is not the main determinant of AML biology (29), however with different approaches.

**Table 1:** Differences on sAML classification within WHO22 and ICC22.

Classification criterion	WHO22	ICC22	Remarks
**Genetic aberrations**	Used for classification; however, secondary myeloid neoplasms have a separate category basing on remote history.	Genetic aberrations are prioritized in defining AML. “Anamnestic qualifiers” may be appointed to any AML diagnosis	ICC22 gives overriding importance to genetic abnormalities.
**Blast threshold for AML diagnosis**	≥20% blasts (except for molecular-defining cytogenetics abnormalities)	≥10% blasts for certain genetic abnormalities	The blast threshold is inconsistent between ICC22 and WHO22 classifications.
**Therapy-related AML**	Considered a separate entity basing on remote history	Used as a diagnostic qualifier.	ICC22 shifts focus from clinical history to genetic profile.
**Germline-predisposition AML**	Considered a separate entity basing on remote history/genetics	Used as a diagnostic qualifier.	There are minor differences in genes acknowledged to give germline predisposition; genes causing some complex syndromes are not accounted in WHO22 classification.
**Myelodysplasia-related AML**	Used for classification within AML; AML with myelodysplasia-related abnormalities is classified as an AML subcategory (MR-AML)	Used as a diagnostic qualifier. Biological entities of myelodysplasia-related cytogenetics and myelodysplasia-related mutations are accounted as MDS/AML and AML subcategories.	Reflects ICC22’s emphasis on genetic features over clinical history.
**AML evolution of other chronic myeloid disorders**	It is maintained in the chronic myeloproliferative neoplasms category unless evolutions come from MDS/MPN; in this case, it is classified as MR-AML.	Used as a diagnostic qualifier. Biological entities may be assigned irrespective to remote history if patient has criteria.	WHO22 classification tends to maintain evolution of non-MDS chronic diseases within the chronic disease category.

At our center, we did not manage to create (another) personalized classification that includes most relevant aspects of both WHO22 and ICC22. Our approach is to classify all the patients according to WHO 2016 (12), WHO22, and ICC22 classifications in our reports, and we will presumably maintain this approach up to the moment in which undisputable agreement will be found with an appropriate scientific discussion. Of note, as long as drug prescription is of concern, we should remember that all the demonstration of benefits that we had in clinical trials are based on older classifications (e.g., WHO 2016 for gilteritinib, venetoclax+azacitidine, ivosidenib+azacitidine, CPX351; older classifications for all the other drugs). The benefit of interventions in 2022 classifications’ newly defined AMLs should be a matter of research instead of an Aristotelian assumption.

## 3 Genetic of sAML

**Figure 1:**
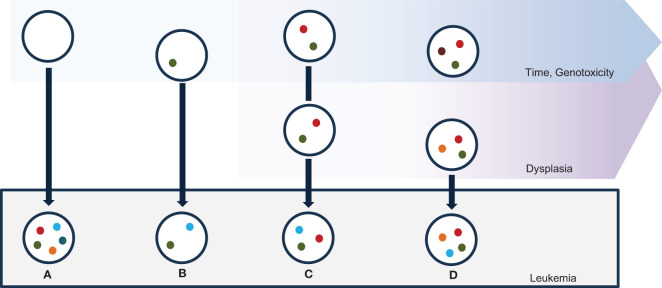
Mechanisms of sAML ontogenesis. Myeloid mutations are spontaneously acquired in the bone marrow with ageing, due to natural genotoxicities; specific genotoxic events may exacerbate the rate of mutations. Leukemic transformation happens toward different putative mechanisms. **(A)** sAML alterations may be acquired via a one-step catastrophic event in an elsewhere normal stem cell. **(B)** A cell containing a single leukemia-predisposing alteration may evolve via a leukemia-promoting “second hit”. **(C)** Time-dependent accumulation of several myeloid alterations may cause sAML via acquisition of a leukemia-promoting event. **(D)** A leukemia-promoting event may be acquired in a subclone in an oligoclonal bone-marrow with plenty of myeloid genes mutations.

The emergence of secondary sAML presents a complex interplay of cellular dynamics and oncogenetic events. Unlike primary AML, which arises *de novo*, sAML develops because of a predefined event, manifesting diverse pathways of transformation (**Figure 1**). Understanding the multifaceted nature of sAML genesis is essential as it engenders distinct hierarchies within cellular populations, influencing disease progression, therapeutic response, and clinical outcomes (30). In this chapter, we delve into the varied mechanisms underlying the evolution of sAML, exploring how these disparate pathways shape the cellular landscape and delineate hierarchical structures within the leukemic population.

### 3.1 AML deriving from catastrophic events

After chemotherapy, or due to other predisposing factors, genomic events may characterize at once. We previously described that complex karyotype alterations and catastrophic events may be acquired in a single step instead of via stepwise acquisition of multiple alterations (31–33). Overall, the acquisition of complex genomic events or localized hypermutations is rare in AML (34, 35). Complex genome events include chromothripsis, chromoanasynthesis, and chromoplexy. Chromothripsis involves massive rearrangements within localized chromosomal regions, caused by catastrophic events leading to micronuclei formation or DNA bridge fragmentation. In AML, it is strongly linked to TP53 mutation and development of marker chromosomes, thus conferring a very adverse prognosis (36, 37). Chromoanasynthesis results from defective DNA plication, leading to regional copy-number gains and insertion of short nucleotide sequences; chromoplexy is characterized by chains of translocations involving multiple chromosomes, which arises from DNA double-strand breaks. Although their impact on AML is less clear due to limited studies. Exposure to chemotherapy and radiation therapy can also select for hematopoietic clones harboring mutations associated with CHIP, thereby increasing the risk of developing therapy-related myeloid neoplasms (t-MNs). This selection occurs because the mutations confer a survival advantage to the clones in the face of genotoxic stress, leading to their preferential expansion (14, 15, 38, 39).

### 3.2 AML deriving from clonal hematopoiesis

AML can arise after an acquired event in a clonal hematopoiesis context. CHIP specifically refers to somatic mutations accumulating in hematopoietic stem cells over time in the absence of any cytopenia or myeloid malignancy. This phenomenon is due to aging bone marrow, in which Darwinian-selected dominant clones acquire proliferative capacity, primarily leading to inflammation and systemic distress and consequently to overt disease (40–42). CHIP-related mutations can be found in platelets and red-blood cell progenitors, but also in immune cells like monocytes, granulocytes, and lymphocytes; mutations affect immune function and increase inflammation (41, 43–45).

The development of myeloid neoplasms from CHIP is influenced by various molecular mechanisms:

- Genetic mutations: Mutations in genes like PPM1D, ASXL1, TP53, IDH1, and IDH2 increase the risk of myeloid neoplasms by promoting clonal expansion and dysregulation of hematopoietic differentiation (46–48).
- Epigenetic alterations: Epigenetic modifiers play a significant role in CHIP progression to myeloid malignancies by affecting gene expression and cellular identity (13, 45, 49, 50).
- Cellular stress responses: Cellular stresses, including DNA damage and inflammatory signals, can select for clones with mutations that confer a survival advantage, thereby increasing the risk of transformation. CHIP progression can also be influenced by external factors such as environmental exposures, systemic inflammation, and the bone marrow microenvironment (51–53).

These factors can create selective pressures that favor the expansion of mutated clones or contribute to the acquisition of additional mutations that drive progression toward malignancy. Of note, AML may branch from previous myeloid disorders; in these circumstances, bone marrow populations are more likely to be polyclonal and with multiple mutations and chromosome alterations, since the genotoxic stimuli are extreme in a pathological condition (11, 54–56). There is evidence that complex clonality and harboring more than one myelodysplasia-related mutation impact outcome (57, 58).

### 3.3 Relevance of specific mutations

**Figure 2:**
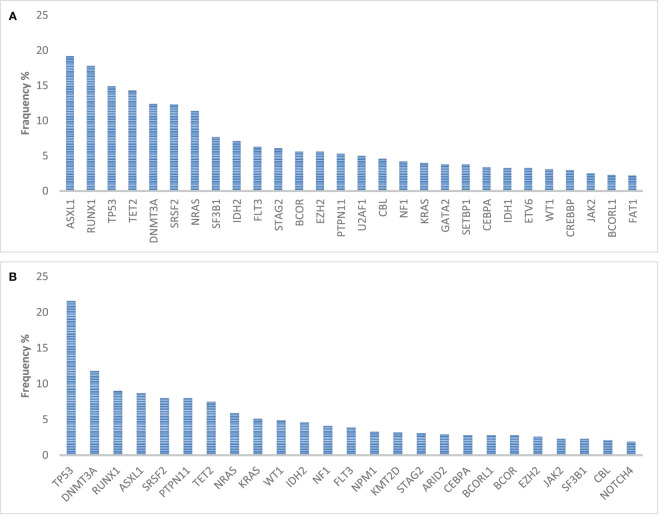
Incidence of mutations in genes relevant for hematopoiesis and in 1,154 myelodysplasia-related AML **(A)** patients and in 389 therapy-related AML patients **(B)**. Query on the Genie 15.0 dataset of 7,156 leukemia patients (59).

Of relevance, druggable mutations are underrepresented in sAML (**Figure 2**), ranging from 10% to 16% of the total population. Thus, therapies that include target drugs are nowadays significant only for a small fraction of the total population.

Overall, prominent characteristics of sAML genetics are the presence of complex genome events, and the presence of mutations that disrupt mechanisms of transcriptional regulation, chromatin organization, RNA splicing, and DNA repair; however, fusions and mutations specifically reported as consequence of chemotherapy exposure and secondary activating mutations are common (**Table 2**). Patterns of evolution will be a significant determinant for future studies, since accumulating evidence suggests differences in disease evolution dynamics (66, 67).

**Table 2:** Cytogenetic changes and gene mutations common in sAML.

Altered pathway/feature	Mutations/cytogenetic changes	Description
**Cytogenetic abnormality**	Complex karyotype, unbalanced clonal abnormalities (e.g., 5q deletion, monosomy 7, 17p deletion, 12p deletion, isochromosome 17q) *	Includes complex karyotypes and specific deletions or unbalanced translocations affecting various chromosomes, indicative of genomic instability and associated with poor prognosis.
	Recurrent translocations (KMT2A rearrangements and MECOM rearrangements) **	Atypical rearrangements of KMT2A or MECOM are frequently reported in patient exposed to chemotherapy (60, 61).
	Marker chromosomes **	Marker chromosomes arise from mitotic crisis and are hallmarks of macroscopic genomic stress (36).
**Transcriptional regulation**	RUNX1 *, ASXL1, BCOR, WT1 (62) **	Mutations disrupt transcription factors and corepressors, altering gene expression crucial for hematopoietic differentiation and the suppression of leukemogenesis.
**Epigenetic regulation and chromatin modification**	ASXL1, EZH2, IDH1/2 **, TET2 **	Involves alterations in chromatin remodeling and histone methylation, impacting gene expression and contributing to the progression of myeloid malignancies.
**RNA splicing**	SF3B1, SRSF2, U2AF1, ZRSR2	Mutations in components of the splicing machinery lead to aberrant RNA splicing, frequently associated with myelodysplastic syndromes and progression to sAML.
**Cohesin complex component**	STAG2	Mutation in STAG2 disrupts the cohesin complex, affecting cell division and genomic stability, which is crucial in the pathogenesis of several cancers including sAML.
**DNA damage response** **	TP53, PPM1D (63)	Mutations in DNA damage response proteins confer resistance to apoptosis and are crucial for cancer persistence.
**Signaling pathways** **	FLT3, NRAS, KRAS, CBL, CSF3R, PTPN11	Activating mutations in signaling pathways are often a second event in AML ontogenesis. These mutations are frequently reported in secondary leukemia, especially leukemia arising after myeloproliferative or myeloproliferative/myelodysplastic neoplasms (42, 64, 65).

*Full agreement has not been reached between WHO22 and ICC22 on cytogenetics changes or genetic mutations that define MR-AML. For the interest of the reader, WHO22 and ICC22 definitions of MR-AML and key differences are reported in **Supplementary Table S2**.

**These changes do not define MR-AML; however, they are common in sAML.

#### 3.3.1 TP53 mutations

TP53 mutations are emerging as a highly unmet need and have high prevalence in sAML (approximately 22% of therapy related AML and 15% of myelodysplasia related AML). TP53 mutation remains the most significant challenge to manage this disease, identifying a subgroup of patients with particular short survival with the main available treatments. Specifically, the poor median survival that is expected for patients receiving intensive chemotherapy or hypomethylating agents alone (raging between 5 and 7 months) was not ameliorated by CPX-351 5.0 months or venetoclax + azacytidine; merely considering the complete remission rate, venetoclax seemed to confer a slightly amelioration of probability of remission (from roughly 30% to 50%) that was not translated into better survival in current experiences, whereas CPX-351 did not augment remission probability compared with standard 3 + 7 (68–71). A possible explanation of this resistance may rely in the fact that there is no gain of function of TP53 protein that is essential for AML cell survival; TP53 loss generates an identical phenotype when compared with TP53 mutations and the molecular alteration stay—in fact—undruggable (72). In TP53 mutant AML, the search of innovative strategies of transcriptional reprogramming, as tamibarotene, or of immunological activity remain pivotal to pave the way for innovative treatments (73, 74).

## 4 Therapeutic strategies

Nowadays, sAML presents unique therapeutic challenges compared with primary AML, and outcomes remain poor (75). Treatment modalities for secondary AML are tailored to the individual’s specific situation, considering factors such as the patient’s age, general health, and cytogenetic and molecular abnormalities. Allogeneic stem cell transplantation (allo-SCT) remains the cornerstone for potentially curative therapy, especially for those in remission and with suitable donors.

### 4.1 Induction therapy

Liposomal formulation of cytarabine and daunorubicin (CPX-351) represent the cornerstone of sAML induction therapy. In a seminal study, CPX-351 demonstrated to improve prognosis over standard “3 + 7” chemotherapy in chemotherapy-suitable patients over the age of 60 (76). Notably, long-term survival was obtained by 18% of CPX-treated patients vs. 8% of 3 + 7 treated patients (77). Even with similar complete remission rates, CPX diminished toxicity significantly augment the probability of transition to allo-SCT and consequently the access to the only known procedure that is potentially curative in this patient population (78, 79). Furthermore, CPX-351 was comparable with “high intensity” FLAG-Ida in a population of cytogenetically defined high-risk AML and MDS, with an advantage of survival in patients with myelodysplasia-related mutation (80). Consequently, the administration of CPX-351 is suggested in any sAML suitable patient. Of note, venetoclax added to chemotherapy may abrogate the prognostic impact of some myelodysplasia-related mutations (81); however, randomized comparisons between CPX-351 and venetoclax-containing inductions are not available. Feasibility studies that combine venetoclax and CPX-351 (NCT03629171) and midostaurin and CPX-351 (NCT04075747) are ongoing.

In the AML18 trial, a percentage of clinically defined and mutational defined sAML patients were treated with gemtuzumab in combination with daunorubicin and cytarabine. The trial confirmed a low likelihood of response to this therapy for sAML, including a lower probability of MRD negative CR when compared with *de novo* patients. Furthermore, a signal toward increased platelet toxicity was noted for patients with myelodysplasia mutations; analysis of OS according sAML status is pending (82). Overall, these data confirmed a poor applicability of gemtuzumab ozogamicin in the sAML population (83, 84). In the setting of CPX-351 or venetoclax + FLAG-Ida unavailability, standard induction regiment should be preferred over the addition of gemtuzumab.

The use of hypomethylating agents, eventually with the addition of venetoclax (VEN), is gaining interest as a bridge to transplant, particularly in this population. In a randomized study that compared a general population of AML older than 60 years 3 + 7 and 10-day decitabine, a trend toward a better functioning was noted for decitabine in sAML (85). Furthermore, the ALFA group generated a prognostic classifier that identifies patients over the age of 60 that were biologically unfit for chemotherapy, thus paving the way for a personalized, low-intensity-based, induction approach (86). Studies that will compare VEN and hypomethylating agents (HMA) with the standard-of-care induction chemotherapy are highly warranted, but up to the results of these studies, a low-intensity approach as induction in sAML is hard to be suggested in patients suitable for chemotherapy; however, boundaries of clinical fitness should be carefully evaluated, especially in this population.

### 4.2 Transplant

The role of allo-SCT in the treatment of sAML is pivotal, serving as a cornerstone for achieving a potentially curative outcome (87). Thus, Allo-SCT is uniformly suggested in the first complete remission in any suitable patient (9). This therapeutic strategy is particularly significant in the context of secondary AML due to the disease’s complex nature, arising from prior hematologic disorders or the aftermath of cytotoxic therapies. The efficacy of allo-SCT hinges on its ability to provide a healthy, donor-derived hematopoietic system capable of eradicating malignant cells, a process greatly facilitated by the graft-versus-leukemia effect (88–90). The selection of allo-SCT as a treatment modality requires a nuanced approach, considering the patient’s remission status, general health, and the presence of suitable donors, which are critical for the transplant’s success and the minimization of associated risks, such as graft-versus-host disease. Thus, allo-SCT represents a vital, albeit complex, therapeutic option within the arsenal against secondary AML, necessitating careful patient selection and optimization of timing to maximize its curative potential while minimizing risks (91). Of note, sAML exhibits an inferior prognosis when transplanted in first complete remission compared with *de novo* patients. This disparity is quantitatively supported by augmented cumulative incidence of relapse and non-relapse mortality and may be independent from measurable residual disease and demand for better and innovative approaches in this population (5, 92, 93). Since measurable residual disease may not have a pivotal role in selecting low-relapse risk patients within sAML, myeloablative conditioning should be suggested in any suitable patient (94–96).

### 4.3 Treatment of unfit patients

The treatment of unfit patients is mainly based on the venetoclax and azacytidine (AZA) combination, which proved superiority compared with single agent azacytidine (97). Within unfit sAML, myelodysplasia-related mutations do not significantly confer a negative prognosis during VEN+AZA therapy (98).

Of note, also glasdegib and cytarabine combination seems to be particularly effective in sAML and may be less toxic if compared with VEN+HMA (99, 100). However, since there is no study comparing VEN+AZA with glasdegib+cytarabine, and since the control arm in the phase 3 study of glasdegib is low-dose cytarabine (101), the use of glasdegib is often restricted to frail patients that are supposed to not tolerate venetoclax, or to patients that were previously exposed to azacytidine and/or venetoclax (e.g., for myelodysplasia).

Decitabine was entitled an interesting approach in the population, mainly after the preliminary demonstration that 10-day exposure might circumvent the negative impact of TP53 mutations (102). However, a small experience on a patient population particularly enriched of sAML (and genetically defined MR-AML) failed in demonstrating any superiority of prolonged exposure (103). Up to now, there is no clinical evidence that suggests decitabine administration over other treatments in this population.

### 4.4 Boundaries of unfitness and biological fitness

The boundaries between clinical and biological fitness are particularly significant in sAML population, especially for patients that are between 60 and 75 years old. As an example, preliminary results from a large study do not demonstrate significant advantage for standard chemotherapy over less intensive 10-day decitabine as long as patients were transplant candidates (85).

Commonly acknowledged and well-validated clinical criteria are based on organ dysfunction unrelated to leukemia and on general clinical conditions (104, 105); strictly, any choice about drug prescriptions should be based on these criteria, since there were the criteria used for assessment of patients during drug experimentation and on which are based clinical-graded evidence that we have. Geriatric assessment and comorbidity-based scores remain an important research effort but are not commonly adopted today for AML treatment decisions (106–109).

Biological fitness, on the other hand, refers to the aspects of an individual’s disease and overall health that make them suitable candidates for intensive therapies. This includes good performance status and adequate organ function, the presence of only limited and manageable comorbidities, and favorable genetic and molecular profiles (that predict good probability of response to chemotherapy). The more interesting approach in personalizing the treatment plan currently comes from the ALFA group, as previously mentioned (86). Of note, the chemotherapy of choice in the population was i.v. administered, 3 + 7 like standard chemotherapy, and thus did not include CPX-351, which has a completely different safety profile. In the near future, approaches that try to delineate between unfitness and biological fitness in AML will be crucial for decision-making in the treatment strategy, guiding the use of intensive treatment modalities versus less intensive or supportive care approaches. This could ensure that treatment plans are both effective and manageable for the patient, ultimately aiming to achieve the best possible outcomes with an emphasis on the patient’s overall health and quality of life.

### 4.5 Treatment of relapse

Treatment of relapse is particularly complicated in sAML since most of the patients have primary resistance or post-transplant relapse. Furthermore, target therapies are hardly applicable to this population, due to the incidence of druggable mutations (**Figure 2**). Our opinion is to prefer a target treatment whenever it is available, eventually in combination with VEN (110–112). All the other patients should be candidate to a clinical trial, due to the lack of effective treatments and the poor outcome. Whenever a clinical trial is not available, the most intriguing alternatives are represented by HMA-based therapies, eventually combined with an immunological therapy such as donor lymphocyte infusion (113–120). In patients with previous exposure to HMA and VEN, the space for non-experimental treatments is unfortunately limited, and this population represents the most significant with a spot for active treatment (121, 122). At the other end of the spectrum, we do not see as for now any reason to avoid a VEN+HMA salvage to patients that were exposed to chemotherapy+VEN, due to the well-known metabolic synergism of VEN+HMA combination, an effect that is different from the apoptosis priming that we experience administering VEN with chemotherapy (123, 124).

## 5 Conclusions

In conclusion, this review has delved into the intricate landscape of sAML, illuminating the progress in understanding its pathogenesis, and the significant strides made in its treatment modalities. Within sAML, genetics seems to maintain a pivotal role if compared with anamnestic classifiers; thus, karyotype alterations and mutations should cover the main role in refining the prognostication and personalizing therapeutic strategies. However, challenges persist, including resistance mechanisms and the need for strategies to manage relapsed or refractory disease. Future research directions should focus on unraveling these complexities, enhancing the efficacy of existing therapies, and exploring innovative treatment combinations (125, 126). Immune-system-based therapies as checkpoint inhibitors, immune-cell engagers, and engineered cell therapies have been invested to be the gamechanger in the field, especially in some subtypes of sAML (e.g., TP53 mutant AML). Unfortunately, we do not manage at the current date a mature approach. Even if allogeneic transplant outcomes strongly suggest that immunotherapy is the way to follow, the keystone remains hard to be found due to antigen and microenvironment-related limitations. Hopefully, research efforts will be better directed in low-burden or measurable residual disease positive AML soon (127). The goal remains to translate these scientific advancements into clinically meaningful benefits, ultimately improving survival rates and quality of life for sAML patients.

## Author contributions

GM: Conceptualization, Data curation, Formal analysis, Methodology, Supervision, Validation, Visualization, Writing – original draft, Writing – review & editing. MR: Supervision, Validation, Visualization, Writing – original draft, Writing – review & editing. BZ: Validation, Visualization, Writing – original draft, Writing – review & editing. IZ: Writing – original draft, Writing – review & editing. DN: Writing – original draft, Writing – review & editing. AM: Writing – original draft, Writing – review & editing. SR: Validation, Visualization, Writing – original draft, Writing – review & editing. FL: Supervision, Writing – original draft, Writing – review & editing.

## Supplementary material

Supplementary Table 1 Germline predisposition to leukemia according to WHO22 and ICC22 s, specifically reporting classification terms.

Supplementary Table 2 Definition of MR AML by genetic and cytogenetic changes according to WHO22 and ICC22 classifications.
