# Supplementary material for: Novel insights and therapeutic approaches in secondary AML
Source: Front Oncol. 2024 Jul 29;14:1400461. doi: 10.3389/fonc.2024.1400461 (PMC11317385; doi:10.3389/fonc.2024.1400461)
Supplement: Supplementary Table 1 — Germline predisposition to leukemia according to WHO22 and ICC22 s, specifically reporting classification terms. [file Table_1.pdf]

Supplementary

Table S1: Germline predisposition to leukemia according to WHO22 and ICC22 s, specifically reporting classification terms

Disease/phenotype	Genetic	WHO22	ICC22
**Myeloid neoplasms with germline predisposition without a pre-existing platelet disorder or organ dysfunction**	CEBPA	Germline CEBPA P/LP variant (CEBPA-associated familial AML)	Germline predisposition due to CEBPA P/LP variants
	DDX41	Germline DDX41 P/LP variant	Germline predisposition due to DDX41 P/LP variants
	TP53	Germline TP53 P/LP varianta (Li-Fraumeni syndrome)	Li-Fraumeni syndrome
**Myeloid neoplasms with germline predisposition and pre-existing platelet disorder**	RUNX1	Germline RUNX1 P/LP varianta (familial platelet disorder with associated myeloid malignancy, FPD-MM)	Germline predisposition due to RUNX1 P/LP variants
	ANKRD26	Germline ANKRD26 P/LP varianta (Thrombocytopenia 2)	Germline predisposition due to ANKRD26 P/LP variants
	ETV6	Germline ETV6 P/LP varianta (Thrombocytopenia 5)	Germline predisposition due to ETV6 P/LP variants
**Myeloid neoplasms with germline predisposition and potential organ dysfunction**	GATA2	Germline GATA2 P/LP variant (GATA2-deficiency)	Germline predisposition due to GATA2 P/LP variants
	ELANE, G6PC3GFI1, HAX1, JAGN, TCRG1, VPS45A	Bone marrow failure syndromes, Severe congenital neutropenia (SCN)	Severe congenital neutropenia
	SBDS (> 90%), DNAJC21, EFL1, SRP54	Bone marrow failure syndromes, Shwachman-Diamond syndrome (SDS)	Shwachman-Diamond syndrome

	FANC A-W	Bone marrow failure syndromes, Fanconi anaemia (FA)	Fanconi anemia
	ACD, CTC1, DKC1, MDM4, RTEL1, TERC, TERT, TINF2, ACD, NHP2, NOP10, NPM1, PARN, WRAP53, RPA1, Apollo	Telomere biology disorders	Telomere biology disorders/short telomere syndromes
	CBL	RASopathies (Neurofibromatosis type 1, CBL syndrome, Noonan syndrome or Noonan syndrome-like disordersa,b)	CBL syndrome
	PTPN11, NRAS, KRAS	“”	Noonan syndrome
	NF1	“”	Neurofibromatosis type I
	+21	Down syndromea,b	
	SAMD9	Germline *SAMD9* P/LP variant (MIRAGE Syndrome)	Germline predisposition due to SAMD9 P/LP variants
	SAMD9L	Germline *SAMD9L* P/LP variant (SAMD9L-related Ataxia Pancytopenia Syndrome)c	Germline predisposition due to SAMD9L P/LP variants
	BLM	Biallelic germline *BLM* P/LP variant (Bloom syndrome)	Bloom syndrome
**Germline predisposition genes causing multiple cancer types including myeloid neoplasms**	CHEK2	Not reported	Germline predisposition due to CHEK2 P/LP variants
	MPL	Not reported	Germline predisposition due to MPL P/LP variants
	RECQL4	Not reported	Germline predisposition due

			to RECQL4 P/LP variants
	BRCA1 or BRCA2	Not reported	Hereditary breast and ovarian cancer
	MLH1, MSH2, MSH6, PMS2	Not reported	Lynch syndrome
	NBN	Not reported	Nijmegen breakage syndrome
	WAS	Not reported	Wiskott-Aldrich syndrome
**Emerging disorders**	CSF3R	Not reported	Germline predisposition due to CSF3R P/LP variants
	ERCC6L2	Not reported	Germline predisposition due to ERCC6L2 P/LP variants
	JAK2	Not reported	Germline predisposition due to JAK2 P/LP variants
	MBD4	Not reported	Germline predisposition due to MBD4 P/LP variants
	MECOM/EVI1	Not reported	Germline predisposition due to MECOM/EVI1 P/LP variants
	NPM1	Not reported	Germline predisposition due to NPM1 P/LP variants
	RBBP6	Not reported	Germline predisposition due to RBBP6 P/LP variants
	SRP72	Not reported	Germline predisposition due to SRP72 P/LP variants
	TET2	Not reported	Germline predisposition due

to TET2 P/LP variants

Table S2: definition of MR AML by genetic and cytogenetic changes according to WHO22 and ICC22 classifications

Feature	WHO22	ICC22	Key Differences
**Complex Karyotype**	≥3 abnormalities not involving specific recurring translocations	≥3 unrelated chromosome abnormalities excluding hyperdiploid karyotypes with multiple trisomies without structural abnormalities	ICC22 explicitly excludes hyperdiploid karyotypes with trisomies; WHO22 does not specify this exclusion.
**Chromosome 5 Abnormalities**	5q deletion or loss of 5q due to unbalanced translocation	del(5q)/t(5q)/add(5q)	Both classifications recognize 5q abnormalities, but ICC22 includes add(5q).
**Chromosome 7 Abnormalities**	Monosomy 7, 7q deletion, or loss of 7q due to unbalanced translocation	−7/del(7q)	-
**Chromosome 8 Abnormalities**	-	+8	WHO22 does not specifically include +8 as a defining feature under MR AML.
**Chromosome 11**	11q deletion	-	ICC22 does not include 11q deletion
**Chromosome 12 Abnormalities**	12p deletion or loss of 12p due to unbalanced translocation	del(12p)/t(12p)/(add)(12p)	
**Chromosome 13 Abnormalities**	Monosomy 13 or 13q deletion		ICC22 does not include 13 ore 13q deletion
**Chromosome 17 Abnormalities**	17p deletion or loss of 17p due to unbalanced translocation, Isochromosome 17q	i(17q), −17/add(17p) or del(17p)	-
**Chromosome 20 abnormalities**	-	del(20q)	WHO22 does not include del(20q)
**Chromosome X Abnormalities**	idic(X)(q13)	-	ICC22 does not include idic(X)(q13)
**Gene Mutations**	ASXL1, BCOR, EZH2, SF3B1, SRSF2, STAG2, U2AF1, ZRSR2	ASXL1, BCOR, EZH2, RUNX1, SF3B1, SRSF2, STAG2, U2AF1, ZRSR2	ICC22 includes RUNX1 as a defining mutation for MR AML, which WHO22 does not list under the defining mutations for MR AML.
